# Particle Interactions Mediated by Dynamical Networks: Assessment of Macroscopic Descriptions

**DOI:** 10.1007/s00332-017-9408-z

**Published:** 2017-08-17

**Authors:** J. Barré, J. A. Carrillo, P. Degond, D. Peurichard, E. Zatorska

**Affiliations:** 1 Laboratoire MAPMO, CNRS, UMR 7349, Fédération Denis Poisson, FR 2964, Université d’Orléans, B.P. 6759, 45067 Orléans Cedex 2, France; 2 Institut Universitaire de France, Paris, France; 3 Department of Mathematics, Imperial College London, London, SW7 2AZ UK; 4 Faculty of Mathematics, University of Vienna, Oskar-Morgenstern Platz 1, 1090 Vienna, Austria

**Keywords:** Dynamical networks, Cross-links, Microscopic model, Kinetic equation, Diffusion approximation, Mean-field limit, Aggregation–diffusion equation, Phase transitions, Fourier analysis, Bifurcations, 82C21, 82C22, 82C31, 65T50, 65L07, 74G15

## Abstract

We provide a numerical study of the macroscopic model of Barré et al. (Multiscale Model Simul, 2017, to appear) derived from an agent-based model for a system of particles interacting through a dynamical network of links. Assuming that the network remodeling process is very fast, the macroscopic model takes the form of a single aggregation–diffusion equation for the density of particles. The theoretical study of the macroscopic model gives precise criteria for the phase transitions of the steady states, and in the one-dimensional case, we show numerically that the stationary solutions of the microscopic model undergo the same phase transitions and bifurcation types as the macroscopic model. In the two-dimensional case, we show that the numerical simulations of the macroscopic model are in excellent agreement with the predicted theoretical values. This study provides a partial validation of the formal derivation of the macroscopic model from a microscopic formulation and shows that the former is a consistent approximation of an underlying particle dynamics, making it a powerful tool for the modeling of dynamical networks at a large scale.

## Introduction

Complex networks are of significant interest in many fields of life and social sciences. These systems are composed of a large number of agents interacting through local interactions, and self-organizing to reach large-scale functional structures. Examples of systems involving highly dynamical networks include neural networks, biological fiber networks such as connective tissues, vascular or neural networks, ant trails, polymers, economic interactions. (Boissard et al. 2013; Mogilner and Edelstein-Keshet 2007; DiDonna and Levine 2006; Broedersz et al. 2010). These networks often offer great plasticity by their ability to break and reform connections, giving to the system the ability to change shape and adapt to different situations (Boissard et al. 2013; Chaudury et al. 2007). For example, the biochemical reactions in a cell involve proteins—DNA, RNA, gene promotors linking/unlinking to create/break large structures–complex of molecules (Kupiec 1997). Because of their paramount importance in biological functions or social organizations, understanding the properties of such complex systems is of great interest. However, they are challenging to model due to the large amount of components and interactions (chemical, biological, social, etc). Due to their simplicity and flexibility, individual-based models are a natural framework to study complex systems. They describe the behavior of each agent and its interaction with the surrounding agents over time, offering a description of the system at the microscopic scale (see, e.g., Barré et al. 2017; Boissard et al. 2013; Degond et al. 2016). However, these models are computationally expensive and are not suited for the study of large systems. To study the systems at a macroscopic scale, mean-field or continuous models are often preferred. These last models describe the evolution in time of averaged quantities such as agent density and mean orientation. As a drawback, these last models lose the information at the individual level. In order to overcome this weakness of the continuous models, a possible route is to derive a macroscopic model from an agent-based formulation and to compare the obtained systems, as was done in, e.g., Barré et al. (2017), Boissard et al. (2013), Degond et al. (2016) for particle interactions mediated by dynamical networks.

A first step in this direction has been made in Barré et al. (2017), following the earlier work (Degond et al. 2016). In this work, the derivation of a macroscopic model for particles interacting through a dynamical network of links is performed. The microscopic model describes the evolution in time of point particles which interact with their close neighbors via local cross-links modeled by springs that are randomly created and destructed. In the mean-field limit, assuming large number of particles and links as well as propagation of chaos, the corresponding kinetic system consists of two equations: for the individual particle distribution function and for the link densities. The link density distribution provides a statistical description of the network connectivity which turns out to be quite flexible and easily generalizable to other types of complex networks.

In the large-scale limit and in the regime where link creation/destruction frequency is very large, it was shown in Barré et al. (2017), following Degond et al. (2016), that the link density distribution becomes a local function of the particle distribution density. The latter evolves on the slow time scale through an aggregation–diffusion equation. Such equations are encountered in many physical systems featuring collective behavior of animals, chemotaxis models, etc. (Topaz et al. 2006; Blanchet et al. 2006; Carrillo et al. 2010; Golestanian 2012; Kolokolnikov et al. 2013) and references therein. The difference between this macroscopic model and the aggregation–diffusion equations studied in the literature Carrillo et al. (2003), Topaz et al. (2006), Bertozzi et al. (2009) lies in the fact that the interaction potential has compact support. As a result, this model has a rich behavior such as metastability in the case of the whole space (Burger et al. 2014; Evers and Kolokolnikov 2016) and exhibits phase transitions in the periodic setting as functions of the diffusion coefficient, the interaction range of the potential, and the links equilibrium length (Barré et al. 2017). By performing the weakly nonlinear stability analysis of the spatially homogeneous steady states, it is possible to characterize the type of bifurcations appearing at the instability onset (Barré et al. 2017). We refer to Barbaro and Degond (2014), Chayes and Panferov (2010), Degond et al. (2015), Barbaro et al. (2016) for related collective dynamics problems showing phase transitions.

If numerous macroscopic models for dynamical networks have been proposed in the literature, most of them are based on phenomenological considerations and very few have been linked to an agent-based dynamics. On the contrary, the macroscopic model proposed in Barré et al. (2017) and its precursor (Degond et al. 2016) have been derived via a formal mean-field limit from an underlying particle dynamics (see also Degond et al. 2014). However, because the derivation performed in Barré et al. (2017) is still formal, its numerical validation as the limit of the microscopic model as well as the persistence of the phase transitions at the micro- and macroscopic level as predicted by the weakly nonlinear analysis in Barré et al. (2017) needs to be assessed. This is the goal of the present work.

More precisely, we show that the macroscopic model indeed provides a consistent approximation of the underlying agent-based model for dynamical networks, by confronting numerical simulations of both the micro- and macromodels. Moreover, we numerically check that the microscopic system undergoes in one-dimensional a phase transition depicted by the values obtained for the limiting macroscopic aggregation–diffusion equation. Furthermore, we numerically validate the weakly nonlinear analysis in Barré et al. (2017) for the type of bifurcation in the two-dimensional setting, where simulations for the microscopic model are prohibitively expensive.

In summary, the main contributions of this work are as follows: (i) It provides a numerical validation of the macroscopic model in 1D as its derivation from the microscopic one in Barré et al. (2017) was only formal. It justifies its further use in 2D where the microscopic model is too computationally intensive. (ii) It also provides the experimental validation of the formal bifurcation analysis for the macroscopic model performed in Barré et al. (2017). In particular, it confirms that the two types of bifurcations revealed in Barré et al. (2017)—subcritical and supercritical—do actually occur. (iii) Finally, it shows that this bifurcation structure is indeed relevant for the microscopic model, for which no theoretical analysis exists to date.

The paper is organized as follows. In Sect. 2, we present the microscopic model and sketch the derivation of the kinetic and macroscopic models from the agent-based formulation. In Sect. 3, we focus on the one-dimensional case: We first summarize the theoretical results on the stability of homogeneous steady states of the macroscopic model from Barré et al. (2017) and show that both the macroscopic and microscopic simulations are in good agreement with the theoretical predictions made by nonlinear analysis of the macroscopic model. We then compare the profiles of the steady states between the microscopic and macroscopic simulations and show that the two formulations are in very good agreement, also in terms of phase transitions. Finally, in Sect. 4 we provide a numerical study of the two-dimensional case for the macroscopic model. The two-dimensional numerical simulations on the macroscopic model are able to numerically capture the subcritical and supercritical transitions as predicted theoretically. Because of the computational cost of the microscopic model, the macroscopic model is not only very competitive and efficient in order to detect phase transitions, but also it is almost the only feasible choice showing the main advantage of the limiting kinetic procedure.

## Derivation of the Macroscopic Model

### Microscopic Model

**Fig. 1:**
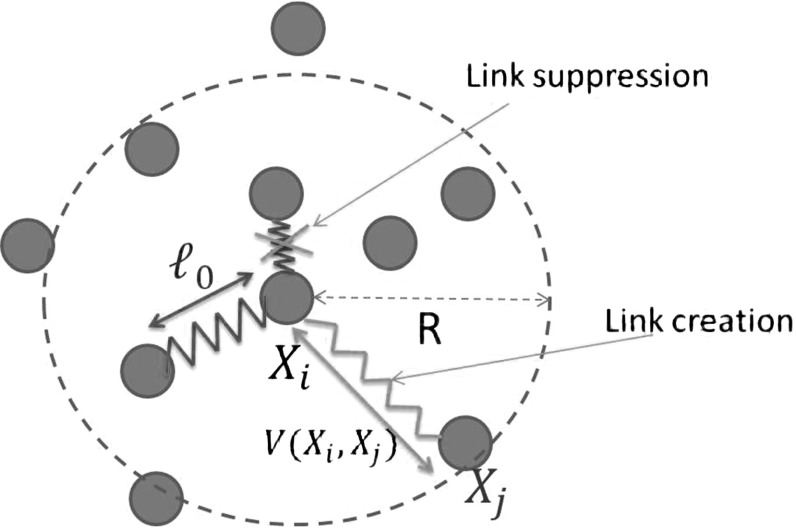
Particles interacting through a network of links seen as springs of equilibrium length *l*. The detection zone for linking to close neighbors is a disk of radius *R*. Link suppression/creation is supposed to be random in time

The two-dimensional microscopic model features *N* particles located at points $X_i \in \Omega , i\in [1,N]$ linking/unlinking—dynamically in time—to their neighbors which are located in a ball of radius *R* from their center. The link creation and suppression are supposed to follow Poisson processes in time, of frequencies $\nu _f^N$ and $\nu ^N_d$, respectively (see Fig. 1).

Each link is supposed to act as a spring by generating a pairwise potential

1 $$\begin{aligned} \tilde{V}(X_i,X_j) = U(|X_i-X_j|) = \frac{\kappa }{2}(|X_i - X_j| - \ell )^2, \end{aligned}$$

where $\kappa$ is the intensity of the spring force and $\ell$ the equilibrium length of the spring. We define the total energy of the system *W* related to the maintenance of the links:

2 $$\begin{aligned} W = \sum _{k=1}^K \tilde{V}(X_{i(k)},X_{j(k)}), \end{aligned}$$

where *i*(*k*)*andj*(*k*) denote the indexes of particles connected by the link *k*. Particle motion between two linking/unlinking events is then supposed to occur in the steepest descent direction to this energy, in the so-called overdamped regime:

3 $$\begin{aligned} \mathrm{d}X_i = - \mu \nabla _{X_i} W \mathrm{d}t + \sqrt{2D}\mathrm{d}B_i, \end{aligned}$$

for $i \in [1,N]$ and where $B_i$ is a two-dimensional Brownian motion $B_i = (B_i^1,B_i^2)$ with diffusion coefficient $D>0$ and $\mu >0$ is the mobility coefficient.

### Kinetic Model

To perform the mean-field limit, following Barré et al. (2017) and Degond et al. (2016), we define the one-particle distribution of the *N* particles, $f^N(x,t)$, and the link distribution of the *K* links, $g^K(x_1,x_2,t)$. Postulating the existence of the following limits:

$$\begin{aligned} f(x,t)= & {} \underset{N \rightarrow \infty }{\lim }{f^N}, \quad g(x_1,x_2,t) = \underset{K \rightarrow \infty }{\lim }{g^K},\\ \nu _f= & {} \underset{N \rightarrow \infty }{\lim }{\nu _f^N(N-1)}, \quad \nu _d = \underset{N \rightarrow \infty }{\lim }{\nu _d^N}, \quad \xi = \underset{K,N \rightarrow \infty }{\lim }{\frac{K}{N}}, \end{aligned}$$

the kinetic system reads:

4 $$\begin{aligned} \partial _t f(x,t) = D \Delta _x f(x,t) + 2\mu \xi \nabla _x \cdot F(x,t) \end{aligned}$$

5 $$\begin{aligned} \partial _t g(x_1,x_2,t)&= D (\Delta _{x_1} g + \Delta _{x_2} g) \nonumber \\&\quad + 2\mu \xi \left[ \nabla _{x_1} \cdot \left( \frac{g(x_1,x_2)}{f(x_1)} F(x_1,t) \right) + \nabla _{x_2} \cdot \left( \frac{g(x_1,x_2)}{f(x_2)} F(x_2,t)\right) \right] \nonumber \\&\quad + \frac{\nu _f}{2\xi } f(x_1,t)f(x_2,t) \chi _{|x_1-x_2|\le R} - \nu _d g(x_1,x_2,t), \end{aligned}$$

where we have postulated that the distribution of pairs of particles reduces to $f(x_1,t)f(x_2,t)$, and

$$\begin{aligned} F(x,t) = \int g(x,y,t)\nabla _{x_1} \tilde{V}(x,y) \mathrm{d}x\mathrm{d}y. \end{aligned}$$

We refer the reader to Barré et al. (2017) for details on the mean-field limit.

In the equation for the limit distribution of particles (4), the first term on the right-hand side is a linear diffusion term which is an effect of the random motion of the particles on the microscopic level. The second term is the attractive–repulsive part due to a spring-like force between the particles that are linked. Its counterpart appears in the equation for the limit distribution of links (5). This equation has also a diffusion part and the production term (the last two terms on the right-hand side) which is due linking processes taking place between the particles that are not yet connected, and unlinking processes breaking the existing links.

### Scaling and Macroscopic Model

In this paper, the space and time scales are chosen such that $\mu = 1$ and the variables are scaled such that:

$$\begin{aligned} \tilde{x} = \varepsilon ^{1/2} x,\quad \tilde{t} = \varepsilon t, \quad f^\varepsilon (\tilde{x},\tilde{t}) = \varepsilon ^{-1} f(x,t), \quad g^\varepsilon (\tilde{x}_1,\tilde{x}_2,\tilde{t}) = \varepsilon ^{-2} g(x_1,x_2,t). \end{aligned}$$

The spring force $\kappa$ is supposed to be small, i.e $\tilde{\kappa } = \varepsilon ^{-1} \kappa$, the noise *D* is supposed to be of order 1, and the typical spring length $\ell$ and particle detection distance *R* are supposed to scale as the space variable, i.e., $\tilde{\ell }= \varepsilon ^{1/2} \ell$, $\tilde{R} = \varepsilon ^{1/2} R$. Finally, the main scaling assumption is to consider that the processes of linking and unlinking are very fast, i.e., $\tilde{\nu }_f = \varepsilon ^2 \nu _f, \tilde{\nu }_d = \varepsilon ^2 \nu _d$. In the example of cell dynamics, mentioned in Introduction, see Kupiec (1997), the frequency of linking/unlinking depends on the size of the molecule, and it is a very fast process (order of seconds) while the macroevolution of the cell such as growth of the cell is much slower (order of minutes). For the sake of simplicity, we will consider in this paper that $\frac{\tilde{\nu }_f}{\tilde{\nu }_d} = 1$, and $\tilde{\kappa } = 2$.

For such a scaling, it is shown in Barré et al. (2017) that in the limit $\varepsilon \rightarrow 0$, if we suppose $(f^\varepsilon ,g^\varepsilon ) \rightarrow _{\varepsilon \rightarrow 0} (f, g)$, then:

6a $$\begin{aligned} \partial _t f = D \Delta _x f + \nabla _x \cdot \big (f \; (\nabla _x V*f) \big ) \end{aligned}$$

6b $$\begin{aligned} g(x,y,t) = \frac{\nu _f}{2\xi \nu _d} f(x,t)f(y,t) \chi _{|x-y|\le R}, \end{aligned}$$

for some compactly supported potential *V* such that:

$$\begin{aligned} \nabla _x V = U'(|x|) \chi _{|x|\le R} \frac{x}{|x|} \end{aligned}$$

In this paper, we take $\kappa =2$ in (1); hence, *V* has the form:

7 $$\begin{aligned} { V(x)= \left\{ \begin{array}{ll} (|x|-\ell )^2-(R-\ell )^2,&{}\quad \text{ for } |x|< R,\\ 0&{}\quad \text{ for } |x|\ge R. \end{array} \right. } \end{aligned}$$

It is worthy to mention that in the context of cell dynamics several authors have already advocated for non-local terms of the form in (6) to model cell adhesion, see, for instance, Domschke et al. (2014), Painter et al. (2015), and the references therein. Therefore, we can reinterpret this fast linking/unlinking as a way of modeling cell adhesion at the microscopic level.

In the following, we aim to study theoretically and numerically both the macroscopic model given by Eq. (6) and the corresponding microscopic formulation given by Eq. (3) and rescaled with the scaling introduced in this section. We first focus on the one-dimensional case and we show that the numerical solutions behave as theoretically predicted, and that we obtain—numerically—a very good agreement between the micro- and macroformulations.

## Analysis of the Macroscopic Model in the One-Dimensional Case

### Theoretical Results

In this section, we apply the results of Barré et al. (2017) to the one-dimensional periodic domain $[-L,L]$, to study the stability of stationary solutions of the macroscopic model given by Eqs. (6a) and (7) with $R<L$.

#### Identification of the Stability Region

We first linearize equation (6a) around the constant steady state $\rho ^*=\frac{1}{2L}$, so that the total mass is equal to 1, we denote the perturbation by $\rho$, so we have $f=\rho ^*+\rho$, that satisfies

8 $$\begin{aligned} \partial _{t} \rho ={D\Delta _{x} \rho }+\rho ^*\Delta (V*\rho ), \end{aligned}$$

where *V* is given by (7). We will further decompose *f* into its Fourier modes

$$\begin{aligned} \rho (x)=\sum _{k\in \mathbb {Z}}\hat{\rho }_{k}e_{k},\quad \text{ where } e_{k}=\exp {\left[ i\pi \frac{kx}{L}\right] } \end{aligned}$$

and the Fourier transform is given by

$$\begin{aligned} \hat{\rho }_{k}=\frac{1}{2L}\int _{-L}^{L} \rho (x)e_{-k}\, \mathrm{d}x. \end{aligned}$$

Applying the Fourier transform to (8), a straightforward computation gives

9 $$\begin{aligned} \partial _{t}\hat{\rho }_k=-\left( \frac{\pi k}{L} \right) ^2\left( D+\hat{V}_k \right) \hat{\rho }_k, \end{aligned}$$

where the Fourier modes of the potential *V* are given by

10 $$\begin{aligned} \hat{V}_k&=\frac{2R^3 }{L}\left( -\frac{\sin (z_k)}{z_k^3}+(1-\alpha )\frac{\cos (z_k)}{z_k^2}+\frac{\alpha }{z_k^2} \right) . \end{aligned}$$

Here, we denoted

$$\begin{aligned} \alpha =\frac{\ell }{R},\quad z_k=\frac{\pi R |k|}{L}. \end{aligned}$$

Therefore, the stability of the constant steady state will be ensured if the coefficient in front of $\hat{\rho }_k$ on the r.h.s. of (9) has a non-positive real part for $k=1$. Indeed, as observed in Barré et al. (2017), this condition implies that all the other modes are then also stable. This condition is related to the H-stable/catastrophic behavior of interaction potentials that characterizes the existence of global minimizers of the total potential energy as recently shown in Cañizo et al. (2015), Simione et al. (2015).

#### Characterization of the Bifurcation Type

As shown in Barré et al. (2017), it is possible to distinguish two types of bifurcation as functions of the model parameters. Indeed, if we define:

11a $$\begin{aligned} \lambda =\lambda _{\pm 1}=-\frac{\pi ^2}{L^2}\left( D+\hat{ V}_{1} \right) , \end{aligned}$$

11b $$\begin{aligned} \lambda _{k}=-\frac{\pi ^2k^2}{L^2}\left( D+\hat{V}_{k} \right) , \end{aligned}$$

we have the following proposition (see Barré et al. 2017):

##### Proposition 1

Assume that $\lambda >0$ and $\lambda _{k}<0, \; \forall k \ne \pm 1$. Then,

- if $2\hat{V}_{2} - \hat{V}_{-1}>0$, the steady state exhibits a supercritical bifurcation;
- if $2\hat{V}_{2} - \hat{V}_{-1}<0$, the steady state exhibits a subcritical bifurcation.

Note that the above criterion only involves the potential, but does not involve the parameter *D*, and it only restricts the values of $\alpha$ or $\ell$.

### Numerical Results

#### Choice of Numerical Parameters

In the linearized equation (8), there are four parameters that may vary: *D*, $\ell$, *R*, and *L*. In this part of the paper, we focus on the case where the potential is of comparable range *R* to the size of the domain *L*, and fix the value of the following parameters:

$$\begin{aligned} L=3\quad \text{ and } \quad R=0.75; \end{aligned}$$

therefore, $z_1=\frac{\pi }{4}$. Using (9) and the discussion from the end of Sect. 3.1.1, we can identify the region where the constant steady state is unstable. Computing $\hat{V}_1<0$ and $\hat{V}_1<-D$, respectively, leads to the following restriction for two remaining parameters of the system $\ell$ and *D*:

$$\begin{aligned} \frac{\ell }{0.75}<\alpha _c:=\frac{(4-\pi )(\sqrt{2}+1)}{\pi }\quad \text{ and }\quad (0.75)^2>\frac{D\pi ^2(2+\sqrt{2})}{8\left( \alpha _c-\frac{\ell }{0.75} \right) }, \end{aligned}$$

which allows to approximate the instability region for this particular case as $D<D(\ell )=0.1781(0.4948-\ell )$. We also introduce a notation $\ell _c=R\alpha _c$, which in this case gives $\ell _c=0.4948$. The parameter $\ell _c$ denotes the value of $\ell$ above which the constant steady state is always stable independently of the value of the parameter *D*.

**Fig. 2:**
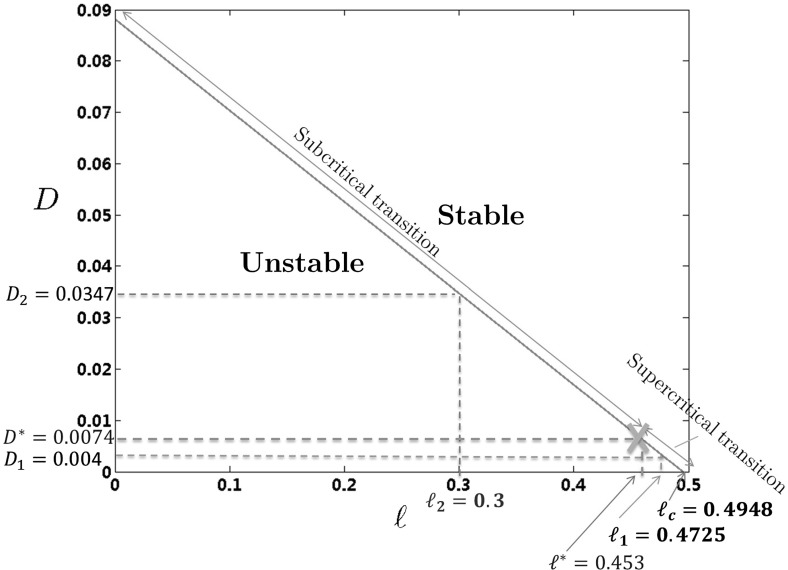
Bifurcation diagram in the one-dimensional case. The critical value for $\ell$, $\ell _c=0.4948$, above which the constant steady state is stable for all values of *D* is indicated in *red*. The change of bifurcation type is located at $(\ell ^*,D^*)=(0.4530,0.0074)$ and indicated in *orange*. For the numerical study, we choose two values of $\ell$: (i) $\ell =\ell _1= 0.4725$, for which a supercritical bifurcation occurs at $D< D_{1}=0.0040$ (indicated in *green*), and (ii) $\ell =\ell _2= 0.3$, for which a subcritical bifurcation occurs at $D< D_{2}=0.0347$ (indicated in *blue*) (Color figure online)

Using (10) and Proposition 1, we check that the bifurcation changes its character for $\ell =\ell ^*$, where $\ell ^*=0.75\frac{(\pi -4)\sqrt{2}+2}{\pi (\sqrt{2}-1)}\approx 0.4530$. Recall that our criterion did not involve the parameter *D*; therefore, the bifurcation is supercritical if only $\ell \in \left( \ell ^*,\ell _c \right) \approx (0.4530, 0.4948)$, and subcritical if $\ell \in (0,l^*)\approx (0, 0.4530)$. The value of parameter *D* corresponding to the instability threshold for $l=l^*\approx 0.4530$ is denoted by $D^*$, and it is equal to 0.0074. All of these parameters are presented in Fig.  2.

##### Remark 1

Choosing *R* comparable to *L* allows us to observe two types of bifurcation: continuous and discontinuous one. It was observed in Barré et al. (2017) that taking $R\ll L$ would cause that for most values of $\ell$ the bifurcation would be subcritical (discontinuous). This effect is captured in Fig. 8, for the two-dimensional case.

#### Macroscopic Model

We now make use of the numerical scheme developed in Carrillo et al. (2015) to analyze the macroscopic equation (6a) with the potential (7) in the unstable regime. The choice of the numerical scheme is due to its free energy decreasing property for equations enjoying a gradient flow structure such as (6a). Keeping this property of gradient flows is of paramount importance in order to compute the right stationary states in the long time asymptotics. In fact, under a suitable CFL condition the scheme is positivity preserving and well balanced, i.e., stationary states are preserved exactly by the scheme.

To check the correctness of the criterion from Proposition 1, we consider two cases corresponding to two different types of bifurcation, as depicted in Fig. 2:

- $\ell _1= 0.4725$ for different values of the noise *D*, where we expect a supercritical (continuous) transition for $D<D_{1} = 0.0040$;
- $\ell _2= 0.3$ for different values of the noise *D*, where we expect a subcritical (discontinuous) transition for $D< D_2 = 0.0347$.

In order to trace the influence of the diffusion on the type of bifurcation, for fixed $\ell _1$, $\ell _2$, we will be looking for the values of diffusion coefficients $D_{1,\lambda }$, $D_{2,\lambda }$ such that

$$\begin{aligned} D_{1,\lambda }\uparrow D_{1}=0.0040,\quad D_{2,\lambda }\uparrow D_{2}=0.0347. \end{aligned}$$

Recall that according to Barré et al. (2017), the parameter $\lambda$ defined in (11a) measures the distance from the instability threshold. We will use this information to determine the values of parameters $D_{1,\lambda }=D_{1,\lambda }(\lambda )$ and $D_{2,\lambda }=D_{2,\lambda }(\lambda )$ computed from (11a). We consider 14 different values for subcritical and supercritical case, as specified in Table 1.

**Table 1:** Table of parameters $D_{1,\lambda }$ (supercritical) and $D_{2,\lambda }$ (subcritical) for the numerical simulations in the macroscopic case with highlighted values corresponding to the phase transition

	$\lambda$	$D_{1,\lambda }$	$D_{2,\lambda }$
1	0.0010	0.0030	0.0338
2	0.0009	0.0031	0.0339
3	0.0008	0.0032	0.0340
4	0.0007	0.0033	0.0340
5	0.0006	0.0034	0.0341
6	0.0005	0.0035	0.0342
7	0.0004	0.0036	0.0343
8	0.0003	0.0037	0.0344
9	0.0002	0.0038	0.0345
10	0.0001	0.0039	0.0346
**11**	**0**	**0.0040**	**0.0347**
12	-0.0001	0.0041	0.0348
13	-0.0002	0.0042	0.0349
14	-0.0003	0.0043	0.0350

Moreover, in Barré et al. (2017) the authors proved that the perturbation $\rho (t)$ of the constant steady state satisfies the following equation

12 $$\begin{aligned} \rho (t,x) = A(t) e_{1}+A^*(t) e_{-1} +A^2(t) h_{2} e_{2} + (A^{*})^2(t) h_{-2} e_{-2} + O((A,A^*)^3), \end{aligned}$$

where

13 $$\begin{aligned} \dot{A}=\lambda A +8\frac{\pi ^4}{L^2}\frac{\hat{V}_{1}}{2\lambda -\lambda _2}\left( 2\hat{V}_{2}-\hat{V}_{1} \right) |A|^2A+ O((A,A^*)^4), \end{aligned}$$

and

$$\begin{aligned} h_{2}= -\frac{4\pi ^2}{L}\frac{\hat{V}_1}{(2\lambda -\lambda _2)},\quad h_{-2}= -\frac{4\pi ^2}{L}\frac{\hat{V}_{-1}}{(2\lambda -\lambda _2)}. \end{aligned}$$

Equation (13) means that for the supercritical bifurcation we can observe a saturation. This means that before stabilizing *A*(*t*) first grows exponentially until the r.h.s. of (13) is equal to zero, i.e., for

14 $$\begin{aligned} |A|=\frac{\sqrt{\lambda }L}{2\sqrt{2}\pi ^2}\sqrt{\frac{2\lambda -\lambda _2}{-\hat{V}_1(2\hat{V}_2-\hat{V}_1)}}. \end{aligned}$$

Using this information to estimate the r.h.s. of (12), we obtain that

15 $$\begin{aligned} |\rho (t,x)|\approx 2|A|+\frac{\lambda L}{\pi ^2(2\hat{V}_2-\hat{V}_1)} = \frac{\sqrt{\lambda }L}{\sqrt{2}\pi ^2}\sqrt{\frac{2\lambda -\lambda _2}{-\hat{V}_1(2\hat{V}_2-\hat{V}_1)}}+\frac{\lambda L}{\pi ^2(2\hat{V}_2-\hat{V}_1)}.\nonumber \\ \end{aligned}$$

This condition gives us the upper estimate for the amplitude of perturbation $\rho$ when the steady state is achieved, that is, after the saturation. The derivation of Proposition 1 in Barré et al. (2017) assumes sufficiently small perturbation of the steady state. Therefore, the initial data for our numerical simulations should be least smaller than the value of |*A*| corresponding to the saturation level. It turns out that |*A*| computed in (14) is always less than $\sqrt{\lambda }$, so the size of initial perturbation of the steady state should be also taken in this regime. If we choose the initial data for the numerical simulations of the supercritical case in this regime, we should see a continuous decay of the saturated amplitude of perturbation to 0, as $\lambda$ decreases. We will perturb the initial data for the subcritical case similarly, showing that even though the smallness restriction is respected, the saturated amplitude of perturbation is a discontinuous function of $\lambda$.

In what follows, we perturb the constant initial condition by the first Fourier mode:

$$\begin{aligned} f_0(x)=\frac{1}{2L}+{\delta (\lambda )} \cos \left( \frac{x\pi }{L} \right) , \end{aligned}$$

with ${\delta (\lambda ) \le \sqrt{\lambda }}$. In the numerical simulations, we consider the case ${\delta }=0.01$. In order to distinguish between the homogeneous steady states (corresponding to the stable regime) and the aggregated steady states (corresponding to the unstable regimes), we compute the following quantifier *Q* on the density profiles of the numerical solutions:

16 $$\begin{aligned} Q = \sqrt{c_1^2 + s_1^2}, \end{aligned}$$

where

$$\begin{aligned} c_1 = \frac{1}{L} \int _{-L}^L f(T_\mathrm{max}, x) \cos \left( \frac{x\pi }{L} \right) \mathrm{d}x,\quad s_1 = \frac{1}{L} \int _{-L}^L f(T_\mathrm{max}, x) \sin \left( \frac{x\pi }{L} \right) \mathrm{d}x, \end{aligned}$$

where $T_\mathrm{max}$ corresponds to the formation of the steady state. Note that (i) if the steady state is homogeneous in space then $Q=0$ and (ii) if *f* is a symmetric function with respect to *x*, then $Q = c_1$.

To estimate $T_\mathrm{max}$, we use the following criterion. From the theory (Carrillo et al. 2003), we know that steady states are positive everywhere and the quantity $\xi =D\log \varrho + V*\varrho$ is equal to some constant *C*. We then compute the distance of $\xi$ from its mean value:

$$\begin{aligned} \xi ^*(t)=\max _{x\in [-L,L]}\left| \xi (t, x)-\frac{1}{2L}\int _{-L}^L\xi (t, x)\ \mathrm{d}x\right| . \end{aligned}$$

**Fig. 3:**
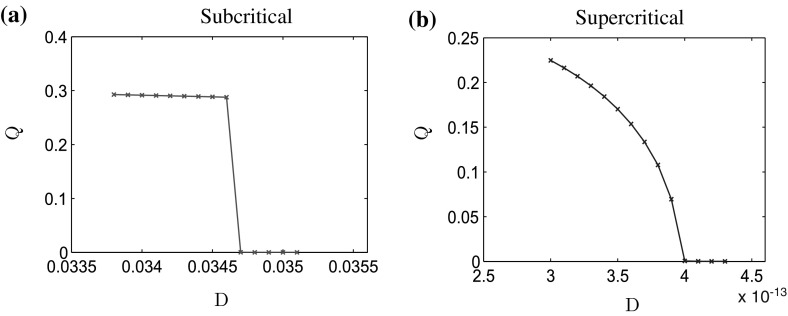
Order parameter *Q* as a function of the diffusion parameter *D* for the macroscopic model for **a** $\ell = 0.3$ (subcritical case) and **b** $\ell =0.4725$ (supercritical case)

The steady state is achieved if $\xi ^*$ is sufficiently close to 0, and in our numerical scheme we continue the computations until $t=T_\mathrm{max}$ for which ${\xi }^*(T_\mathrm{max})<10^{-7}$. The computed values are presented in Tables 5 and 6 in “Appendix 2”. In Fig. 3, we show the values of the order parameter *Q* as a function of the noise intensity *D* for both types of bifurcation.

As shown in Fig. 3, the quantifier *Q* indeed undergoes a discontinuous transition around $D = 0.0347$ for $\ell = 0.3$ (subcritical case, Fig. 3a) and a continuous transition around $D = 0.004$ for $\ell = 0.4725$ (supercritical case, Fig. 3b). These results show that the numerical solutions are in very good agreement with the theoretical predictions.

**Fig. 4:**
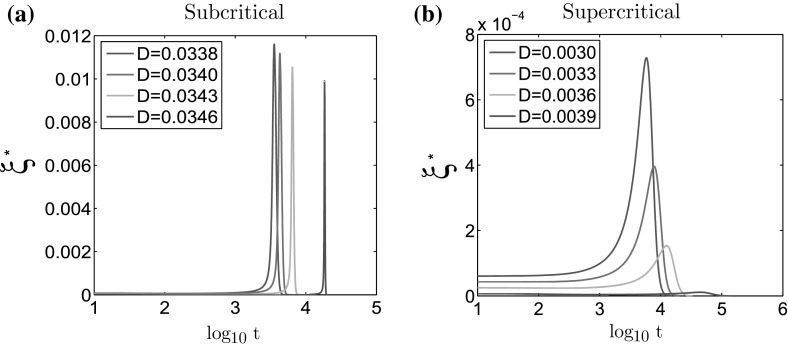
Values of $\xi ^*$ as a function of $\log _{10}t$ computed on the steady states of the macroscopic model for **a** $\ell =0.3$ (subcritical case) and **b** $\ell =0.4725$ (supercritical case)

In order to check the accuracy of our prediction of the value of $T_\mathrm{max}$, we show in Fig.  4 the graph of $\xi ^*(t)$ for several values of *D* in the supercritical and the subcritical cases (see Table 1). As shown in Fig.  4, we observe a very sharp change of $\xi ^*$ for the subcritical bifurcation and much smoother one for the supercritical case. The amplitude change of $\xi ^*$ is also a good indication of the type of bifurcation. As for the order parameter, we see that for the subcritical bifurcation it is on similar level (Fig. 4a) for all values of *D*, while for the supercritical bifurcation it decays to 0 (Fig. 4b). We will use this observation to analyze the results of the two-dimensional simulations later on.

**Fig. 5:**
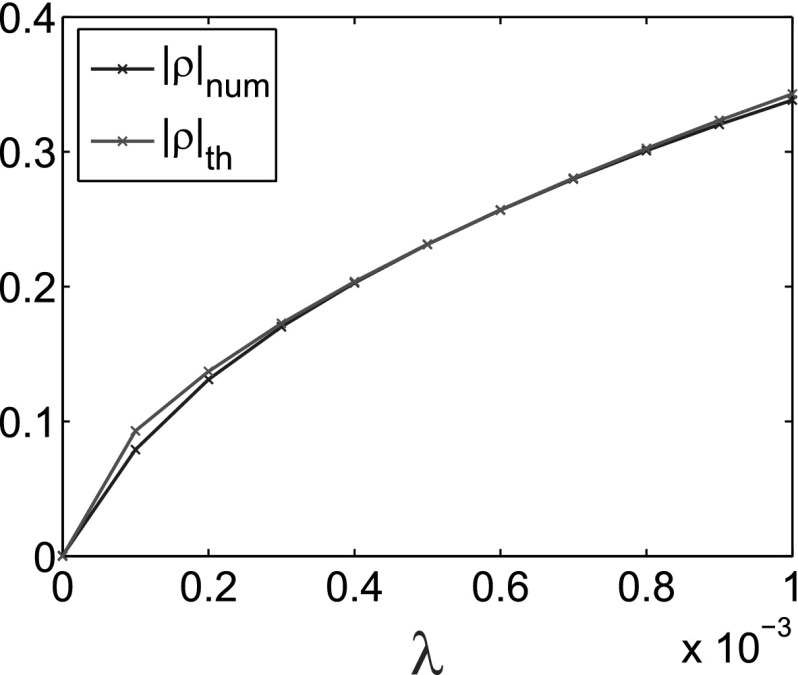
Comparison of theoretical $|\rho |_\mathrm{th}$ with the numerical $|\rho |_\mathrm{num}$

Finally, we can also check how the theoretical prediction of the size of perturbation from (15) is confirmed by our numerical results. For this purpose, we compute the maximum of the perturbation once the steady state is achieved:

$$\begin{aligned} |\rho |_\mathrm{num}=\Vert f(T_\mathrm{max},x)-\rho ^*\Vert _{L^\infty ((-L,L))} \end{aligned}$$

for all the points of supercritical bifurcation. The results are presented in Fig. 5 and in Table 2.

**Table 2:** Theoretical ($|\rho |_\mathrm{th}$) vs numerical ($|\rho |_\mathrm{num}$) values for the size of perturbation

$\lambda$	$|\rho |_\mathrm{num}$	|*A*|	$|\rho |_\mathrm{th}$
0.0010	0.3384	0.1094	0.3428
0.0009	0.3203	0.1058	0.3233
0.0008	0.3008	0.1017	0.3025
0.0007	0.2797	0.0968	0.2805
0.0006	0.2567	0.0912	0.2569
0.0005	0.2312	0.0847	0.2314
0.0004	0.2028	0.0770	0.2036
0.0003	0.1701	0.0678	0.1727
0.0002	0.1311	0.0562	0.1371
0.0001	0.0790	0.0403	0.0930
0	0.0005	0	0

We now aim at performing the same stability analysis on the microscopic model from Sect. 2.1—the starting point of the derivation of the macroscopic model.

#### Microscopic Model

Here, we aim at performing simulations of the microscopic model from Section 2.1, rescaled with the scaling from Section 2.3. After rescaling and if we consider an explicit Euler scheme in time (see “Appendix 1”), we can show that Eq. (3) between time steps $t^n$ and $t^n+\Delta t^n$ reads (in non-dimensionalized variables):

17 $$\begin{aligned} X_i^{n+1} = X_i^n - \nabla _{X_i} W(X^n) \Delta t^n + \sqrt{2{D} \Delta t^n}{\mathcal {N}}(0,1), \end{aligned}$$

where *W* is defined by (2) and ${\mathcal {N}}(0,1)$ is the normal distribution with mean 0 and standard deviation 1. Between two time steps, new links are created between close enough pairs of particles *that are not already linked* with probability $\mathbb {P}_f = 1 - e^{{\nu _f \Delta t^n}/{((N-1)\varepsilon ^2)}}$ and the existing links disappear with probability $\mathbb {P}_d = 1 - e^{-{\nu _d \Delta t^n}/{((N-1)\varepsilon ^2)}}$. Therefore, the rescaled version of the microscopic model features a very fast link creation/destruction rate, as the linking and unlinking frequencies are supposed to be of order $1/\varepsilon ^2$, for small $\varepsilon$. Note also that to capture the right time scale, the time step $\Delta t$ must be decreased with $\varepsilon$, which makes the microscopic model computationally costly for small values of $\varepsilon$. For computation time reasons, we also consider the limiting case $\varepsilon = 0$ of the microscopic model; we can show that it reads:

18 $$\begin{aligned} X_i^{n+1} = X_i^n - \nabla _{X_i} W_0(X^n) \Delta t^n + \sqrt{2{D} \Delta t^n}{\mathcal {N}}(0,1), \end{aligned}$$

where

$$\begin{aligned} W_0(X) = \sum _{i,j |\ |X_i - X_j|\le R} V(X_i,X_j). \end{aligned}$$

Note that in this regime, no fiber link remains and particles interact with all of their close neighbors. The limit $N \rightarrow \infty$ of this limiting microscopic model should exactly correspond to the macroscopic model (6) (see, for instance, Bolley et al. 2011; Fournier et al. 2015; Carrillo et al. 2014; Godinho and Quiñinao 2015 for studies of mean-field limits including, as in the present case, singular forces). If not otherwise stated, the values of the parameters in the microscopic simulations are given in Table 3.

**Table 3:** Table of parameters (non-dimensionalized values)

Parameter	Value	Interpretation
*L*	3	Domain half size
$\delta$	0.1	Maximal step
$T_\mathrm{f}$	20	Final simulation time
$\xi _\mathrm{init}$	0.1	Initial fraction $\frac{K}{N}$
$\nu _d$	1	Unlinking frequency
$\nu _f$	1	Linking frequency
*R*	0.75	Detection radius for creation of links
$\ell$	Adapted	Spring equilibrium length
$\kappa$	2	Spring force between linked fibers
*D*	Adapted	Noise intensity
$\varepsilon$	Adapted	Scaling parameter

As for the macroscopic model, the order of the particle system at equilibrium is measured by the quantifier *Q* defined by Eq. (16), where the integrals are computed using the trapezoidal rule. To compute the density of agents *f*(*x*) in the microscopic simulations, we divide the computational domain $[-L,L]$ into $N_x$ boxes of centers $x_i$ and sizes $\mathrm{d}x = \frac{L}{N_x}$, and for $i= 1\ldots N_x$, we estimate

$$\begin{aligned} {f_i} = \frac{N_i}{2 N L}, \end{aligned}$$

where $f_i=f(x_i)$ and $N_i$ are, respectively, the density and the number of agents whose centers belong to the interval $[-L + (i-1)\mathrm{d}x, -L + i\, \mathrm{d}x]$. Following the analysis of the macroscopic model, we explore the same two cases: $\ell _1= 0.4725$, $D_{1}=0.0040$, and $\ell _2= 0.3$, $D_{2}=0.0347$ to check whether they correspond to the super and subcritical bifurcations, respectively.

**Fig. 6:**
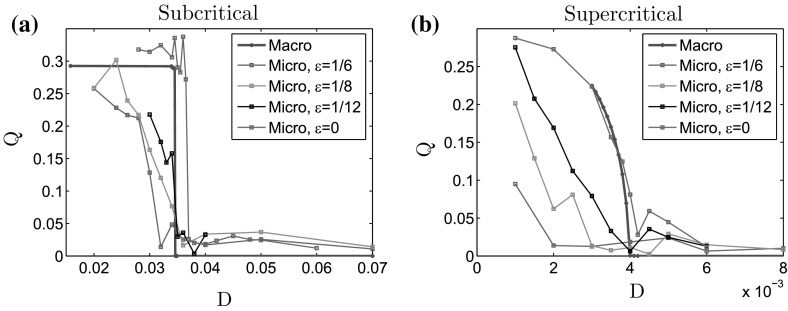
Values of *Q* plotted as functions of the noise intensity *D* computed from the numerical solutions at equilibrium of the macroscopic model (*red curves*), and of the microscopic model for $\varepsilon =1/16$ (*blue curves*), $\varepsilon =1/8$ (*orange curves*), $\varepsilon =1/12$ (*black curves*), and limiting case “$\varepsilon = 0$” [Eq. (18), *green curves*]. **a** For $\ell = 0.3$ (subcritical bifurcation) and **b** for $\ell = 0.4725$ (supercritical bifurcation). For small $\varepsilon$ and these two values of $\ell$, we recover the bifurcation types predicted by the analysis of the macroscopic model. As $\varepsilon$ decreases, the critical values of *D* for which the transitions occur get closer to the ones of the macroscopic model, and in the limiting case “$\varepsilon =0$” in the microscopic model, we obtain a very good agreement between the microscopic and macroscopic models (Color figure online)

In Fig.  6, we show the values of *Q* plotted as functions of the noise intensity *D* computed from the simulations of the scaled microscopic model (17) at equilibrium, for two different values of $\ell$: $\ell = 0.3$ (a), $\ell = 0.4725$ (b), and different values of $\varepsilon$: $\varepsilon = \frac{1}{6}$ (blue curves), $\varepsilon = \frac{1}{8}$ (orange curves), $\varepsilon = \frac{1}{12}$ (black curves), and the limiting case “$\varepsilon = 0$” [Eq. (18), green curves]. For each $\ell$, we superimpose the values of *Q* obtained with the simulations of the macroscopic model (red curves). As expected, we observe subcritical transitions for $\ell = 0.3$ and a supercritical transition for $\ell = 0.4725$. As $\varepsilon$ decreases, the values of the noise intensity *D* for which the transitions occur get closer to the theoretical values predicted by the analysis of the macroscopic model. These results show that the scaled microscopic model has the same properties as the macroscopic one, and that the values of the parameters ($\ell , D$) which correspond to a bifurcation in the steady states tend, as $\varepsilon \rightarrow 0$, to the ones predicted by the analysis of the macroscopic model. Indeed for the limiting case “$\varepsilon =0$” of the microscopic model, we obtain a very good agreement between the micro- and macroformulations, showing that the microscopic model behaves as predicted by the analysis of the macroscopic model.

**Fig. 7:**
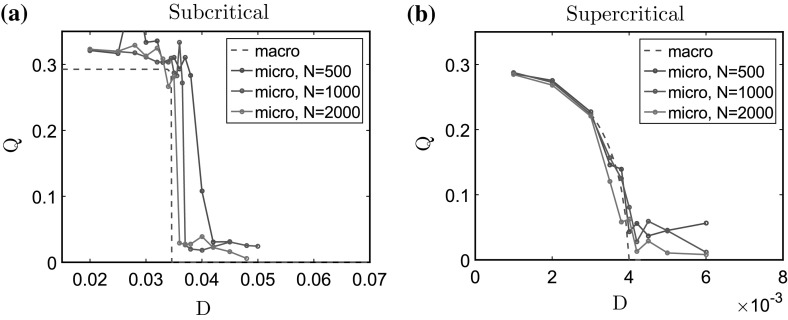
Values of *Q* plotted as functions of the noise intensity *D* computed from the numerical solutions at equilibrium of the macroscopic model (*dashed red curves*), and of the microscopic model for “$\varepsilon =0$” [Eq. (18)], and different values of *N*: $N=500$ (*green curves*), $N=1000$ (*blue curves*), and $N=2000$ (*red curves*). **a** For $\ell = 0.3$ (subcritical bifurcation), **b** for $\ell = 0.4725$ (supercritical bifurcation). For $\varepsilon = 0$, we recover the bifurcation types predicted by the analysis of the macroscopic model. Moreover, as the number of particles *N* increases, the critical values of *D* for which the transitions occur get closer to the ones of the macroscopic model, and for $N=2000$ in the microscopic model, we obtain a very good agreement between the microscopic and macroscopic models (Color figure online)

It is noteworthy that the small differences observed in the values of the transitional *D* (subcritical case, Fig. 6a) can be due to the fact that we use a finite number of $N=1000$ particles for the microscopic simulations, whereas the macroscopic model is in the limit $N\rightarrow \infty$. However, these differences are very small when we consider the limit case $\varepsilon =0$ for the microscopic model. Indeed, determining visually the transitional *D* in the microscopic simulations with neglecting the slight increase appearing after (see Fig. 6b), the relative error between the microscopic and macroscopic transitional *D*, $\frac{|D_\mathrm{mic} - D_\mathrm{mac}|}{D_\mathrm{mac}}$ is $7\%$ for $\ell = 0.3$, and $5\%$ for $\ell = 0.4725$. In order to give a more quantitative analysis on the influence of the number of particles, we show in Fig.  7 the values of *Q* plotted as functions of the noise intensity *D*, for the macroscopic model (dashed red curves), and for the microscopic model with “$\varepsilon =0$” [Eq. (18)] and different number of particles *N*: $N=500$ (green curves), $N=1000$ (blue curves), and $N=2000$ (red curves). Figure 7a shows the case $\ell = 0.3$ (subcritical bifurcation) and (b) the case $\ell = 0.4725$ (supercritical bifurcation). As depicted in Fig. 7, as the number of particle increases, the value of the critical noise intensities $D_c$ get closer to the ones predicted by the macroscopic model for both the subcritical and supercritical transitions. Moreover, the values of *Q* corresponding to space homogeneous equilibria (for $D>D_c$ in both cases) get closer to zero as *N* increases, and its variations after the transitional *D* observed for $N=500$ or $N=1000$ get negligible for $N=2000$. Altogether, these results show that the microscopic model is a good approximation of the macroscopic dynamics when considering a large number of particles and a small value of $\varepsilon$. It is noteworthy that the simulations of the microscopic model become very time-consuming when considering $N=2000$ particles, and we refer the reader to “Computational Aspects of the Micro-and-Macroscopic Models” section of Appendix 1 for a detailed analysis of the computational time.

We now aim at comparing the profiles of the solutions between the microscopic and macroscopic models, to numerically validate the derivation of the macroscopic model from the microscopic dynamics.

#### Comparison of the Density Profiles in the Microscopic and Macroscopic Models

Here, we aim at comparing the profiles of the particle densities of the microscopic model with the ones of the macroscopic model as functions of time. As shown in the previous section, for $\varepsilon$ small enough, we recover the bifurcation and bifurcation types observed from the macroscopic model with the microscopic formulation, with very good quantitative agreement when considering the limiting microscopic model (18) with “$\varepsilon = 0$.” The simulations of the microscopic model are very time-consuming for small values of $\varepsilon$, because we are obliged to consider very small time steps (see “Computational Aspects of the Micro-and-Macroscopic Models” section of Appendix 1). Here, due to computational time constraints, we therefore compare the results of the macroscopic model (6) with $\varepsilon =0$ for which the time step can be taken much larger and independent of $\varepsilon$.

In order to have the same initial condition for both the microscopic and macroscopic models, we initially choose the particle positions for both models such that:

$$\begin{aligned} f_0(x) = \frac{1}{2L} + \delta (\lambda ) \cos \frac{x\pi }{L}. \end{aligned}$$

We send the reader to “Appendix 1” for the numerical method used to set the initial conditions of the microscopic model. Because of the stochastic nature of the model, the microscopic model does not preserve the symmetry of the solution, contrary to the macroscopic model (where noise results in a deterministic diffusion term). To enable the comparison between the macroscopic and microscopic models, we therefore re-center the periodic domain of the microscopic model such that the center of mass of the particles is located at $x=0$ (center of the domain). To this aim, given the set of particles $X_j, j=1 \ldots N$, we reposition all the particles at points $\tilde{X}_j, j=1\ldots N$ such that:

$$\begin{aligned} \tilde{X}_j = {\left\{ \begin{array}{ll} X_j - X_m \quad \text {if } |X_j - X_m|\le L\\ X_j - X_m - 2L \frac{X_j - X_m}{|X_j - X_m|} \quad \text {if } |X_j - X_m|> L, \end{array}\right. } \end{aligned}$$

where $X_m$ is the center of mass computed on a periodic domain:

$$\begin{aligned} X_m =\frac{L}{\pi } \; \hbox {arg} \bigg (\frac{1}{2}\sum _{j=1}^N e^{\frac{i \pi X_j}{L}} \bigg ). \end{aligned}$$

Finally, in order to decrease the noise in the data of the microscopic simulations due to the random processes, the density of particles is computed on a set of several simulations of the microscopic model.

**Fig. 8:**
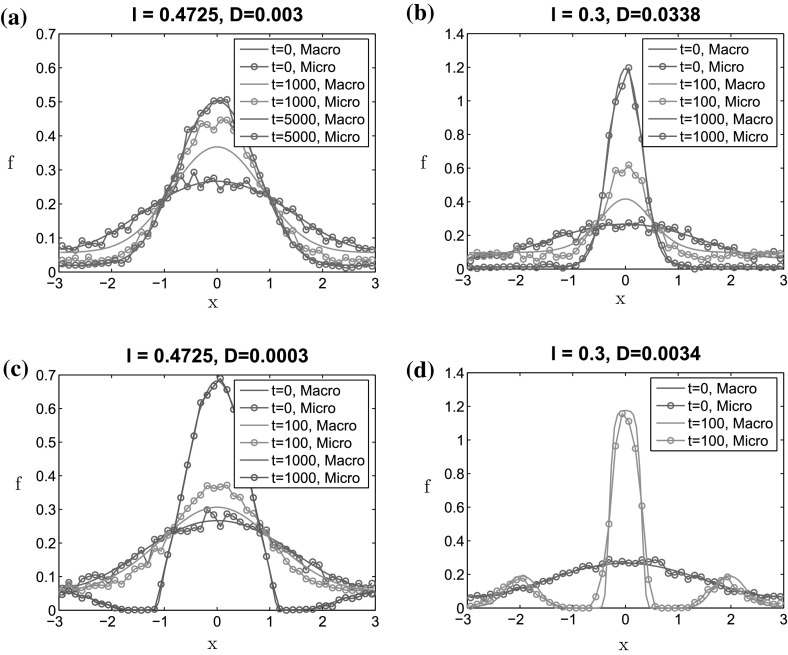
Comparison of the density distributions between the macroscopic model and the microscopic one with “$\varepsilon = 0$,” for different times and two values of $\ell$: $\ell = 0.4725$ (**a**, **c**), and $\ell = 0.3$ (**b**, **d**). *Continuous lines* solution of the macroscopic model, *with circles* solution of the microscopic model with $\varepsilon = 0$, averaged over six simulations. For each value of $\ell$, we consider two different noise intensities *D*: For $\ell = 0.4725$, we use $D = 0.003$ (**a**) and $D=0.0003$ (**c**), and for $\ell =0.3$, we use $D=0.0338$ (**b**) and $D=0.0034$ (**d**)

In Fig.  8, we show the density distributions of the macroscopic model (continuous lines) and of the microscopic one with “$\varepsilon = 0$” (circle markers) at different times, for $\ell = 0.4725$ and $\ell = 0.3$, respectively. For each value of $\ell$, we consider two values for the noise intensity *D*: For $\ell = 0.4725$, we study the cases $D = 0.003$ and $D=0.0003$, and for $\ell = 0.3$, we choose $D = 0.0338$ and $D = 0.0034$. Note that all these values are in the unstable regime.

As shown in Fig.  8, we obtain a very good agreement between the solutions of the macroscopic model and of the microscopic one with “$\varepsilon = 0$.” Close to the transitional *D* (Fig. 8a, b), the particle density converges in time toward a Gaussian-like distribution for both the microscopic and macroscopic models. Note that the microscopic simulations seem to converge in time toward the steady state faster than the macroscopic model (compare the orange curves on the top panels). This change in speed can be due to the fact that the microscopic model features finite number of particles while the macroscopic model is obtained in the limit of infinite number of particles. Therefore, in the macroscopic setting, each particle interacts with many more particles than in the microscopic model, which could result in a delay in the aggregation process.

When far from the transitional *D* in the unstable regime (Fig. 8c, d), one can observe the production of several bumps in the steady state of the particle density. The production of several particle clusters in these regimes shows that the noise triggers particle aggregation. For small noise intensity, local particle aggregates are formed which fail to detect neighboring aggregates. As a result, one can observe several clusters in the steady state, for small enough noise intensities. These bumps are observed for both the microscopic and macroscopic models, showing again a good agreement between the two dynamics.

In the next section, we present a numerical study of the macroscopic model in the two-dimensional case. As mentioned previously, the microscopic model is in very good agreement with the macroscopic dynamics for small values of $\varepsilon$ as in the one-dimensional case. Its simulations are, however, very time-consuming, due to the need of very small time steps (see “Computational Aspects of the Micro-and-Macroscopic Models” section of Appendix 1). As a result, the microscopic model is not suited for the study of very large systems such as the ones considered in the two-dimensional case. We therefore provide a numerical two-dimensional study using the macroscopic model only.

## Analysis of the Macroscopic Model in the Two-Dimensional Case

### Theoretical Results

In this section, we first recall some theoretical results from Barré et al. (2017) for the two-dimensional periodic domain. We will focus on the square periodic domain $[-L,L]\times [-L,L]$, since the rectangular case can be, in agreement with the analysis in Barré et al. (2017), reduced to the one-dimensional case studied above.

The starting point for the phase transition analysis is the linearized equation

$$\begin{aligned} \partial _{t} \rho ={D\Delta _{x} \rho }+\rho ^*\Delta (V*\rho ), \end{aligned}$$

in which the spatially homogeneous distribution $\rho ^*$ is now equal to $\frac{1}{(2L)^2}$. Applying the Fourier transform to this equation, we obtain

$$\begin{aligned} \partial _{t}\hat{\rho }_{k_1,k_2}=-{\pi ^2}\frac{k_1^2+k_2^2}{L^2} \left( D+\hat{ V}_{k_1,k_2} \right) \hat{\rho }_{k_1,k_2}:=\lambda _{k_1,k_2}\hat{\rho }_{k_1,k_2} \end{aligned}$$

and we denote $\lambda _{\pm 1,0}=\lambda _{0,\pm 1}=\lambda$. The Fourier transform of the potential *V* is given by

19 $$\begin{aligned} \hat{ V}_{k_1,k_2}{=}\frac{\pi }{L^2} \left( \frac{\pi R^3 l}{2z_{k_1,k_2}^2}\big [J_1(z_{k_1,k_2})H_0(z_{k_1,k_2}){-}J_0(z_{k_1,k_2})H_1(z_{k_1,k_2})\big ]{-}\frac{R^4}{z_{k_1,k_2}^2}J_2(z_{k_1,k_2}) \right) ,\nonumber \\ \end{aligned}$$

where we denoted

$$\begin{aligned} z_{k_1,k_2}=\frac{\pi R}{L}\sqrt{{k_1^2+k_2^2}}, \end{aligned}$$

and $J_i$ are Bessel function of order *i*

$$\begin{aligned} J_i(x)=\sum _{m=0}^\infty \frac{(-1)^m}{m! \Gamma (m+1+i)}\left( \frac{x}{2} \right) ^{2m+i}, \end{aligned}$$

and $H_i$ are the Struve functions defined by

$$\begin{aligned} H_i(x)=\sum _{m=0}^\infty \frac{(-1)^m}{\Gamma (m+3/2)\Gamma (m+i+3/2)}\left( \frac{x}{2} \right) ^{2m+i+1}. \end{aligned}$$

Again, fixing the ratio $\frac{R}{L}\le 1$, the relation between *D*, *l*, and *R* for the phase transition can be read from the condition $\lambda =0$, which yields

20 $$\begin{aligned} D+\hat{V}_{1,0}=0, \end{aligned}$$

which due to (19) gives

$$\begin{aligned} D\pi +R^2\left( \frac{\pi }{2}\frac{l}{R}\left( J_1(z_{1,0})H_0(z_{1,0})-J_0(z_{1,0})H_1(z_{1,0}) \right) -J_2(z_{1,0}) \right) =0. \end{aligned}$$

The relevant criterion for the type of bifurcation in the two-dimensional case then reads:

#### Proposition 2

Assume *D* is varied such that it crosses the bifurcation point (20), and such that $\lambda _{k_1,k_2}$ remains negative for all $k_1,k_2$ such that $|k_1|+|k_2|>1$, let

$$\begin{aligned} c= \frac{\hat{V}_{1,0}(2\hat{V}_{2,0}-\hat{V}_{-1,0})}{D+\hat{V}_{2,0}},\quad d=-\left| 4 \frac{\hat{V}_{1,0}\hat{V}_{1,1}}{D+\hat{V}_{1,1}}\right| , \end{aligned}$$

then,

- if $c<d$, the constant steady state exhibits a supercritical bifurcation, and
- if $c>d$, the constant steady state exhibits a subcritical bifurcation.

Note that in the two-dimensional case, the bifurcation criterion involves also parameter *D*. On the other hand, the instability threshold *D* is given as a function of $\alpha$ and can be calculated using (20).

### Numerical Results

We first compute the approximate instability regime for the following three cases:

1. For $R/L=1$, $z_{1,0}=\pi$, the constant steady state is unstable for
  $$\begin{aligned} \frac{\ell }{R}<\alpha _c:=0.6620\quad \text{ and }\quad D< 0.2334 R^2{\left( \alpha _c-\frac{\ell }{R} \right) }. \end{aligned}$$
2. For $R/L=1/2$, $z_{1,0}=\frac{\pi }{2}$, the constant steady state is unstable for
  $$\begin{aligned} \frac{\ell }{R}<\alpha _c:=0.7333\quad \text{ and }\quad D< 0.1084 R^2{\left( \alpha _c-\frac{\ell }{R} \right) }. \end{aligned}$$
3. For $R/L=1/4$, $z_{1,0}=\frac{\pi }{4}$, the constant steady state is unstable for
  $$\begin{aligned} \frac{\ell }{R}<\alpha _c:=0.7462\quad \text{ and }\quad D< 0.0312 R^2{\left( \alpha _c-\frac{\ell }{R} \right) }. \end{aligned}$$

Therefore, for $L=3$, the criterion from Proposition 2 gives the following outcomes:

1. For $R/L=1$, the steady state exhibits a supercritical bifurcation for $\alpha \in (0.1016, 0.5818)$ and a subcritical bifurcation for $\alpha \in (0,0.1016)\cup (0.5818,0.6620)$.
2. For $R/L=1/2$, the steady state exhibits only a subcritical bifurcation.
3. For $R/L=1/4$, the steady state exhibits only a subcritical bifurcation.

**Fig. 9:**
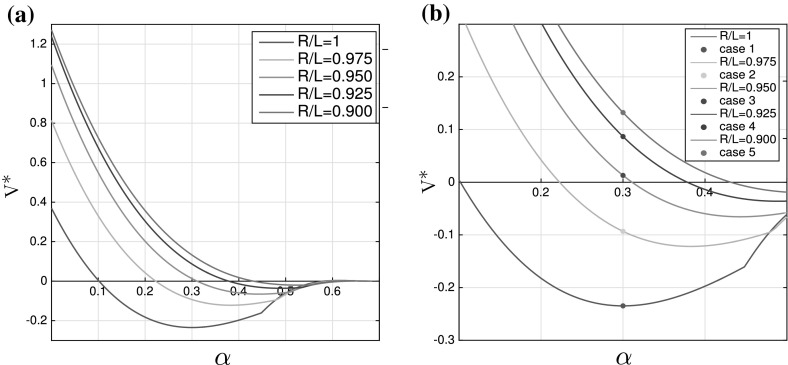
**a** Values of $V^*$ as a function of $\alpha$ for different ratios *R* / *L*. **b** Zoom on the values of $V^*$ around $\alpha =0.3$ for different ratios *R* / *L*, with marker points for $V^*$ at $\alpha =0.3$ (used in the numerical simulations).

This computation confirms the theoretical prediction from Barré et al. (2017) that the smaller $\frac{R}{L}$ is, the more likely it is that the bifurcation is of the subcritical type. The different types of bifurcation happen only for $\frac{R}{L}$ close to 1; otherwise, the bifurcation is always subcritical. To understand this behavior, we compute

$$\begin{aligned} V^*= {\hat{V}_{1,0}(2\hat{V}_{2,0}-\hat{V}_{-1,0})}\left| {D+\hat{V}_{1,1}}\right| +4 \left( D+\hat{V}_{2,0} \right) \left| {\hat{V}_{1,0}\hat{V}_{1,1}}\right| . \end{aligned}$$

From Proposition 2, it follows that if $V^*>0$ the bifurcation is subcritical; otherwise, it is supercritical. We depict the function $V^*(\alpha )$, where $\alpha =\frac{\ell }{R}$ for different values of $\frac{R}{L}\in [0.9,1]$ in Fig. 9a. We see that decreasing the ratio $\frac{R}{L}$ causes that more and more of the graph of $V^*(\alpha )$ lies above 0. This means that for most of the values of $\alpha \in [0,\alpha _c]$ the bifurcation is subcritical.

We will study all of the five cases from Fig. 9b corresponding to different values of $\frac{R}{L}$, but the same value of $\alpha =\frac{\ell }{R}=0.3$. The theoretical prediction is that the first two cases $\frac{R}{L}=1$ and $\frac{R}{L}=0.975$ correspond to a supercritical (continuous) bifurcation while the cases 3–5 correspond to the subcritical (discontinuous) bifurcation.

We perturb the constant initial data as in the one-dimensional case; namely, we take

$$\begin{aligned} f_0(x,y)=\frac{1}{4L^2}+\delta \cos \left( \frac{x\pi }{L} \right) , \end{aligned}$$

with $\delta =0.01$, and similarly to the one-dimensional case we compute the value of the order parameter *Q*

$$\begin{aligned} Q=\frac{1}{2L^2}\int _{-L}^{L}\int _{-L}^{L}f(T_\mathrm{max},x,y)\cos \left( \frac{x\pi }{L} \right) \, \mathrm{d}x\, \mathrm{d}y, \end{aligned}$$

where we used the empirical observation that the steady state is always symmetric with respect to $(x,y)=(0,0)$. For the stopping time criterion, we take the same as in one-dimensional case, namely ${\xi }^\star (T_\mathrm{max})<10^{-7}$.

**Fig. 10:**
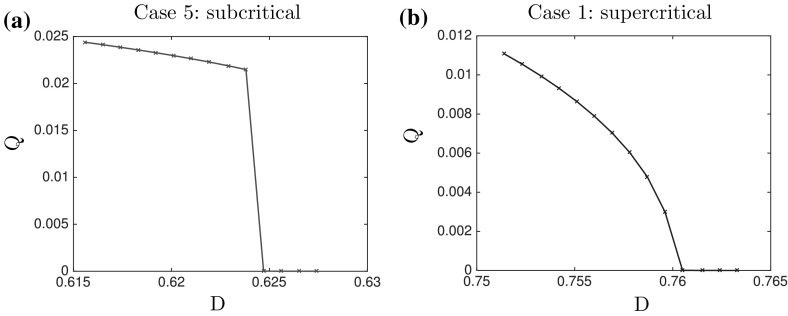
Quantifier *Q* as a function of the diffusion coefficient *D* computed on the steady states of the macroscopic two-dimensional model. These bifurcation diagrams have been generated from the data in Tables 7 and 11 from “Numerical Results for the Two-Dimensional Case” section of Appendix 2. **a** Case 5: subcritical and **b** case 1: supercritical

In Fig. 10, we show the values of the order parameter *Q* as function of the noise intensity *D* for both types of bifurcation for cases 1 and 5, based on Tables 7 and 11 from “Appendix 2”. As shown in Fig. 10, we indeed obtain a supercritical (continuous) transition in the values of *Q* as function of the noise *D* in case 1 (Fig. 10b), while the transition is discontinuous (subcritical) in case 5 (Fig. 10a). These results therefore show that the numerical results are in good agreement with the theoretical predictions and provide a validation of the numerical approximation and simulations of the macroscopic model.

**Fig. 11:**
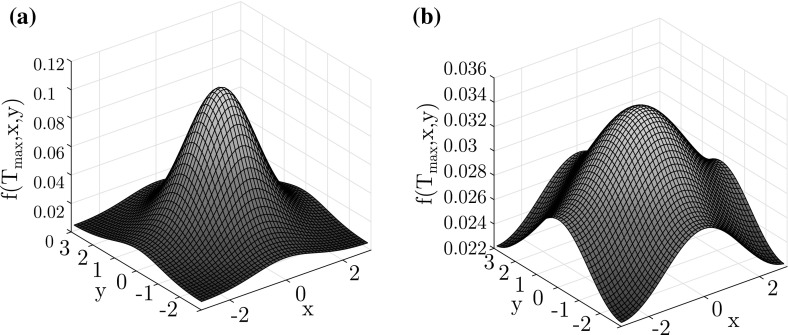
Final density profile in the subcritical case 5 for $D_5=0.6238$ (**a**) and in the supercritical case 1 for $D_1=0.7596$ (**b**). The values of parameters $D_1$ and $D_5$ in both cases correspond to $\lambda =0.001$ in Table 4

The difference between the bifurcation types is also reflected in the amplitude of the steady state. For both types of bifurcation, i.e., for cases 1 and cases 5 we plot the final steady states in Fig. 11. The density profile for the supercritical bifurcation (Fig. 11b) is much lower and rounded than the one for the subcritical bifurcation (Fig. 11a).

**Fig. 12:**
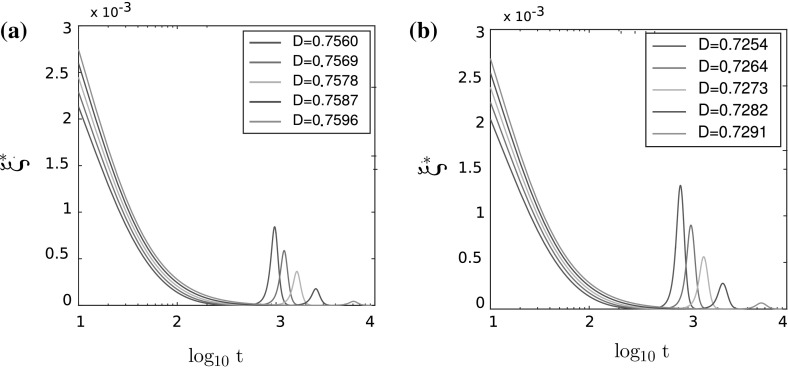
Values of $\xi ^*(\log _{10}t)$ for the supercritical bifurcation for **a** case 1 and **b** case 2

**Fig. 13:**
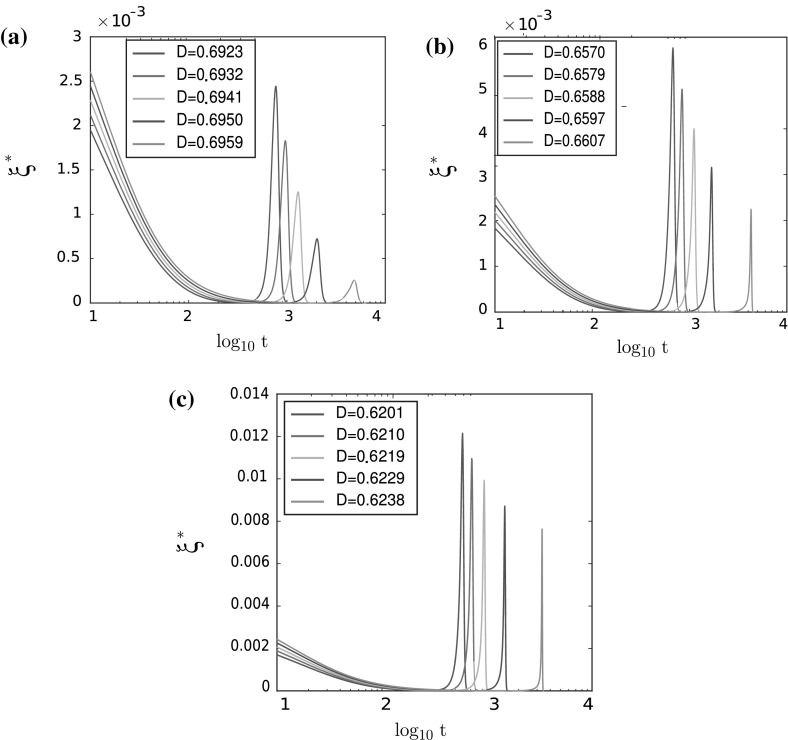
Values of $\xi ^*(\log _{10}t)$ for the subcritical bifurcation for **a** case 3, **b** case 4, and **c** case 5

Moreover, as in the one-dimensional case, we can check that the different bifurcation diagrams correspond to different shapes of $\xi ^*$. In Figs. 12 and 13, we present the graphs of $\xi ^*(t)$ for all five cases depicted in Fig. 9b. For each of the cases, we present the graph of $\xi ^*(t)$ for five different values of diffusion parameter *D* as specified in Table 4.

As shown in Fig. 12, the graph of $\xi ^*(t)$ undergoes smooth changes for the different values of the noise *D*, highlighting a bifurcation of supercritical type. Figure 13 shows that $\xi ^*(t)$ undergoes sharp changes for the different values of the noise *D*, highlighting the subcritical type of bifurcation, as predicted by the theoretical analysis of the macroscopic model in the two-dimensional case. Close to the transition zone (case 3, $\frac{R}{L} = 0.95$, as shown in Fig. 13a), the changes in $\xi ^*$ are smoother than for smaller values of $\frac{R}{L}$ (Fig. 13b, c), but the transition is still subcritical as can be confirmed by the values of order parameter *Q* given in Table 9.

**Table 4:** Table of parameters $D_{1,\lambda }$ and $D_{2,\lambda }$ (supercritical), $D_{3,\lambda }$, $D_{4,\lambda }$, and $D_{5,\lambda }$ (subcritical) for the numerical simulations in two-dimensional case

	$\lambda$	$D_{1,\lambda }$	$D_{2,\lambda }$	$D_{3,\lambda }$	$D_{4,\lambda }$	$D_{5,\lambda }$
1	0.005	0.7560	0.7254	0.6923	0.6570	0.6201
2	0.004	0.7569	0.7264	0.6932	0.6579	0.6210
3	0.003	0.7578	0.7273	0.6941	0.6588	0.6219
4	0.002	0.7587	0.7282	0.6950	0.6597	0.6229
5	0.001	0.7596	0.7291	0.6959	0.6607	0.6238

## Conclusion

In this paper, we have provided a numerical study of a macroscopic model derived from an agent-based formulation for particles interacting through a dynamical network of links. In the one-dimensional case, we were first able to recover numerically the subcritical and supercritical transitions undergone by the steady states of the macroscopic model, in the regime predicted by the theoretical nonlinear analysis of the continuous model. Moreover, the numerical simulations of the rescaled microscopic model revealed the same bifurcations and bifurcation types as obtained with the macroscopic model, with very good precision as $\varepsilon$ goes to zero in the microscopic setting. Finally, when considering the limiting case “$\varepsilon =0$” in the microscopic model, we obtained a very good agreement between the profiles of the solutions of the micro- and macromodels. It is noteworthy that both models also feature the same dynamics in time, with a slight delay in the macroscopic simulations compared to the microscopic dynamics. This delay may be due to the fact that the microscopic simulations are performed with a finite number of particles while the macroscopic model is in the limit of infinite number of individuals. However, as for very small values of $\varepsilon$ the simulations of the microscopic dynamics are very time-consuming, we were not able to extend the numerical study to a higher number of particles.

For the sake of completeness, we finally presented numerical simulations of the model in the two-dimensional case. For computational reasons, we were not able to perform two-dimensional simulations of the microscopic model, and we chose to focus on the macroscopic model. In the two-dimensional case, we were once again able to numerically recover supercritical and subcritical transitions in the steady states, as function of the noise intensity *D*, in the same regime as predicted by the theoretical analysis of the macroscopic model. These results validate the theoretical analysis, the numerical method, and the simulations developed for the macroscopic model.

By providing a numerical comparison between the micro- and macrodynamics, this study shows that the macroscopic model considered in this paper is indeed a relevant tool to model particles interacting through a dynamical network of links. As a main advantage compared to the microscopic formulation, the macroscopic model enables to explore large systems with low computational cost (such as two-dimensional studies) and is therefore believed to be a powerful tool to study network systems on the large scale. Direct perspectives of these works include the derivation of the macroscopic model in a regime of non-instantaneous linking–unlinking of particles. The hope is to understand deeper how the local forces generated by the links are expressed at the macroscopic level. The model could be improved by taking into account other phenomena such as external forces and particle creation/destruction. Finally, rigorously proving the derivation of the macroscopic model from the particle dynamics will be the subject of the future research.

## Figures and Tables

**Fig. 14:**
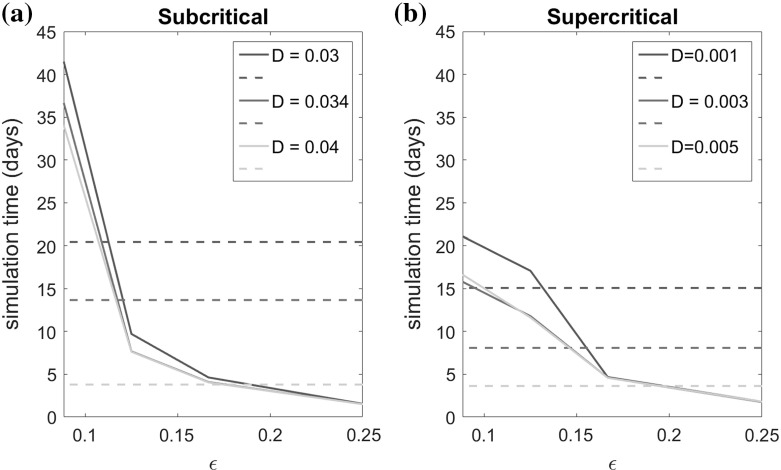
Simulation time of the microscopic model (in days), as function of the scaling parameter $\epsilon$ and for different values of the noise *D*. **a** For the subcritical bifurcation $\ell = 0.3$ and three values of *D*: $D=0.03$ (unstable regime, *blue curve*), $D=0.034$ (close to the bifurcation, *orange curve*), and $D=0.04$ (stable regime, *yellow curve*) and **b** for the supercritical bifurcation $\ell = 0.4725$ and three values of *D*: $D=0.001$ (unstable regime, *blue curve*), $D=0.003$ (close to the bifurcation, *orange curve*), and $D=0.005$ (stable regime, *yellow curve*). Values of the CPU time for “$\epsilon =0$” [Eq. (18)] for the corresponding values of *D* are plotted as *dashed lines* (Color figure online)

**Fig. 15:**
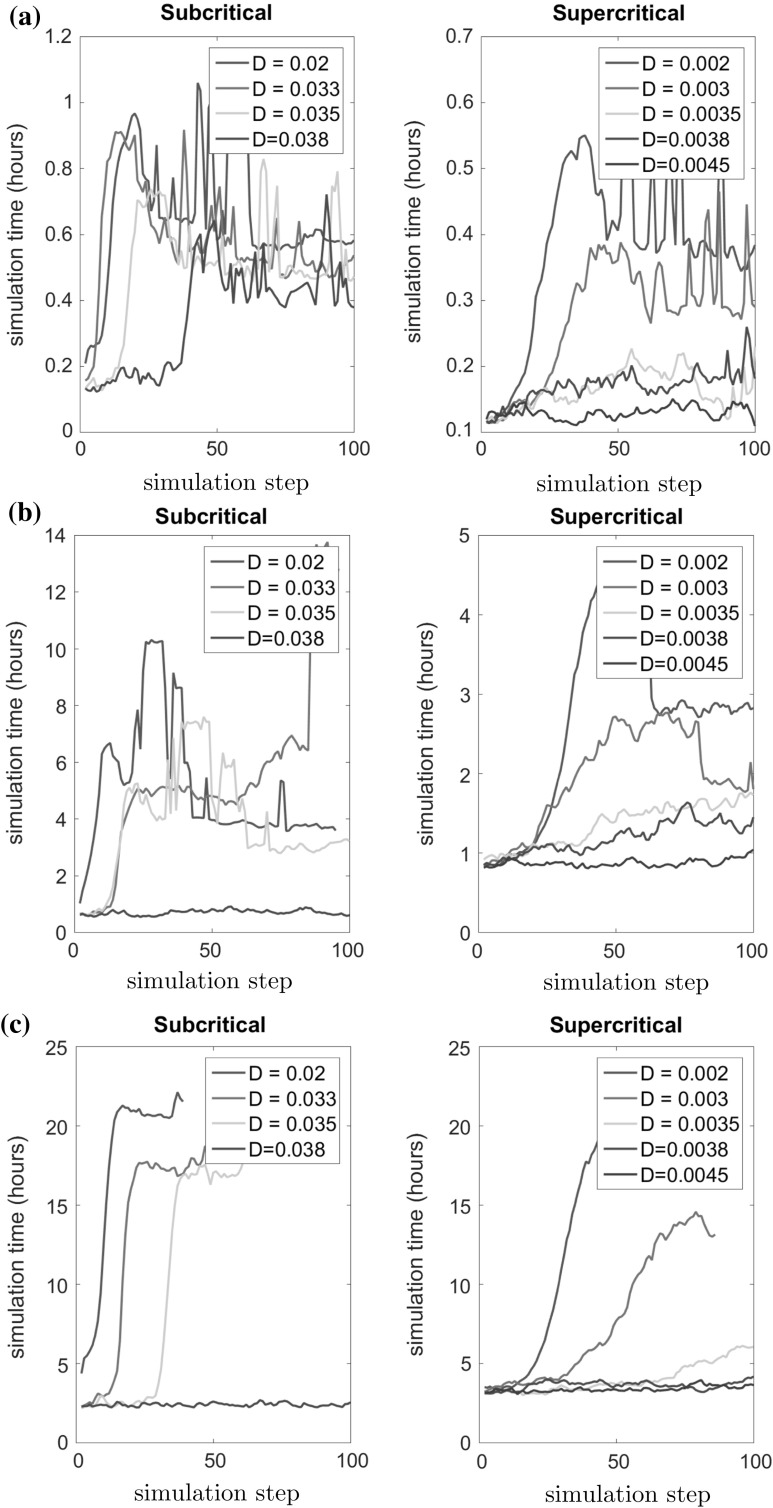
Computational time between two time steps, as function of the simulation time, for the microscopic model with “$\epsilon =0$” and different values of *N*: **a** $N=500$, **b** $N=1000$, and **c** $N=2000$. For the subcritical bifurcation (*left figures*, $\ell = 0.3$), we consider different values of *D*: $D=0.02$ (unstable mode, *blue curves*), $D=0.033$ and $D=0.035$ (close to the transitional *D*, *orange* and *yellow curves*, respectively), and $D=0.038$ (stable mode, *green curves)*. For the supercritical bifurcation (*right figures*), we use $D=0.002$ and $D=0.003$, (unstable modes, *blue* and *orange curves*, respectively), $D=0.0035$ and $D=0.0038$ (close to the transitional *D*, *yellow* and *green curves*, respectively), and $D=0.045$ (stable mode, *red curves*). The CPU time strongly depends on the amount of aggregation of the particles in the simulation, as well as on the total number of particles (Color figure online)

## Appendix 1: On the Micromodel

### The Model

The scaling of Sect. 2.2 obliges us to increase the length of the domain when decreasing $\varepsilon$. For convenience, we rather work with fixing the domain $\Omega$. We therefore use the following scaling:

$$\begin{aligned}&\tilde{x} = x, f^\varepsilon = f, g^\varepsilon = g, \tilde{\kappa } = \varepsilon ^{-1} \kappa , \tilde{D} = \varepsilon D, \tilde{R} = R, \tilde{l} = l, \nonumber \\&\tilde{t} = \varepsilon t, \tilde{\nu }_f = \varepsilon ^2 \nu _f, \tilde{\nu }_d = \varepsilon ^2 \nu _d. \end{aligned}$$

One can check that this scaling after letting $\varepsilon \rightarrow 0$ leads to the same macroscopic system (6). The microscopic model then reads (dropping the tildes for clarity purposes):

$$\begin{aligned} X_i^{n+1} = X_i^n - \nabla _{X_i} W(X^n) \Delta t^n + \sqrt{2{D} \Delta t^n}{\mathcal {N}}(0,1). \end{aligned}$$

Between two time steps, new links are created between close enough pairs of particles *that are not already linked* with probability $\mathbb {P}_f = 1 - e^{-\nu _f^N \Delta t^n}$ and new links disappear with probability $\mathbb {P}_d = 1 - e^{-\nu _d^N \Delta t^n}$, where we denoted $\nu _f^N = \tilde{\nu }_f/((N-1)\varepsilon ^2)$, $\nu _d^N = \tilde{\nu }_d/(N-1)\varepsilon ^2)$. Here, the time step $\Delta t^n$ is chosen such that the particle motion is bounded by the numerical parameter $\delta >0$ and such that $\nu _f^N \Delta t^n<0.1$, $\nu _d^N \Delta t^n <0.1$ (to capture the right time scale). To this purpose, we set:

$$\begin{aligned} \Delta t^n = \min \left( \frac{\delta }{N_{lpf} R \kappa }\, ,\ \frac{0.1}{\max (\nu _f^N,\nu _d^N)} \right) , \end{aligned}$$

where $N_\mathrm{plf}$ is the maximal number of links per fiber. Note that as the links are dynamical, $N_\mathrm{plf}$ might change during the course of the simulation, making the time step dependent on the current step. For the particle simulations, we suppose that the number of particles is large enough so that we can set $\nu _f^N = \frac{\nu _f}{N-1}$ and $\nu _d^N = \nu _d$. Finally, as explained in the main text, we initially choose the particle positions for both models such that:

$$\begin{aligned} f_0(x) = \frac{1}{2L} + \delta (\lambda ) \cos \frac{x\pi }{L}. \end{aligned}$$

For the microscopic model, the initial positions of the particles are set such that given a random position $X \in [-L,L]$ and a random number $\eta _2 \in [-1,1]$, we let the probability of creating a new particle at position $X+ \eta _2 \Delta x - \frac{\Delta x}{2}$ be $\frac{\Delta x}{2L} + \delta (\lambda ) \Delta x \cos \frac{\pi X}{L}$.

### Computational Aspects of the Micro-and-Macroscopic Models

The numerical scheme of the microscopic model is implemented in FORTRAN90. Parallelization is only used when running several simulations for the comparison with the macroscopic model (see Fig. 8) or for the analysis of phase transitions (see Sect. 3.2.3). In Fig. 14, we show the computation time (CPU)—in days—of the microscopic model as function of the scaling parameter $\epsilon$ and for different values of the noise *D*. In Fig. 14a, we consider the case $\ell = 0.3$ (subcritical transition), and Fig. 14b shows the CPU time for $\ell =0.4725$ (supercritical transition). Values of the CPU time for $\epsilon =0$ [Eq. (18)] are plotted as dashed lines. For each $\ell$, we consider three values of *D*: in the unstable regime (blue curves), close to the bifurcation (orange curves), and in the stable regime (yellow curves). As $\epsilon$ decreases, the CPU time increases for both values of $\ell$, and we deduce from Fig. 14a that the CPU time is in $O(\frac{1}{\epsilon ^3})$. As the time step is directly linked to the linking parameters $\nu _f, \nu _d$ of order $O(\frac{1}{\epsilon ^2})$, it is expected that the CPU time is at least in $O(\frac{1}{\epsilon ^2})$. Moreover, the CPU time is larger in the unstable regime than in the stable one (see discussion bellow), and finally, the CFL-type condition of the microscopic model (see previous paragraph) links the time step to the maximal number of links per fiber, also directly linked to the clustering of the particles. Altogether, these effects lead to a CPU time of order $O(\frac{1}{\epsilon ^3})$, dependent on the scaling parameter $\epsilon$ as well as on the value of the statistical quantifier *Q* which measures the “clustering” of the particles (compare Fig. 14 with Fig. 6). Note that in the limit case “$\epsilon = 0$” (dashed lines in Fig. 14), the CPU time is much smaller than for $\epsilon \ne 0$. Indeed, in this limit case there is no more linking dynamics as particles interact with all of their close neighbors. Therefore, in this case, the computational time does not depend on the linking and unlinking frequencies anymore, but still on the quantifier *Q*. Note that these results correspond to a fixed number of particles $N=1000$, but that the CPU time also depends on this parameter.

In order to highlight the dependency of the CPU time on the clustering of particles and on the number of particles, we show in Fig. 15 the evolution of the CPU time—in hours—during the simulations, when considering the microscopic model with “$\epsilon =0$” and different values of *D* and *N*. Contrary to Fig. 14 which shows the total CPU time of a simulation, Fig. 15 shows the computational time between two time steps, as a function of the simulation time. For the subcritical bifurcation (left figures, $\ell = 0.3$), we consider different values of *D*: $D=0.02$ (unstable mode, blue curves), $D=0.033$ and $D=0.035$ (close to the transitional *D*, orange and yellow curves, respectively), and $D=0.038$ (stable mode, green curves). For the supercritical bifurcation (right figures), we use $D=0.002$ and $D=0.003$ (unstable modes, blue and orange curves, respectively), $D=0.0035$ and $D=0.0038$ (close to the transitional *D*, yellow and green curves, respectively), and $D=0.045$ (stable mode, red curves). Figure 15a shows the case $N=500$ particles, and (b) and (c) correspond to $N=1000$ and $N=2000$, respectively. As shown in Fig. 15, the CPU time strongly depends on the amount of aggregation of the particles in the simulation. At the beginning of the simulation, particles are distributed almost homogeneously in space, leading to a small CPU time. As particles start aggregating, one observes an increase in CPU time, by a factor ranging from 3 to 6 as the number of particles increases from 500 to 2000 (Fig. 15a–c). These figures clearly show the simulation time at which particles start aggregating, which depends on the value of *D* as well as on the number of particles and on the regime we consider: Indeed, particles aggregate faster in the subcritical regime than in the supercritical one. The fact that formation of clusters in the unstable regime leads to a larger CPU time than in the stable one is directly linked to the presence of particle aggregates, as each particle interacts with more neighbors in the clustered case than in the homogeneous one. Altogether, these numerical results show that the microscopic model becomes very time-consuming when considering small values of $\epsilon$ and in the unstable regime or even when considering large number of particles. Our simulations reached 40 days for $\epsilon = \frac{1}{12}$ and $D = 0.03$ in the subcritical case ($\ell = 0.3$), whereas the CPU times for the macroscopic model ranged from few minutes to few hours. These results highlight the need for a continuous formalism when studying systems with large number of particles.

**Table 5:** Supercritical bifurcation in the one-dimensional case

$l_1=0.4725$	$D_{1,\lambda }$	$\lambda$	$T_\mathrm{max}$	*Q*
Super-1	0.0030	0.0010	1.52e$+$4	0.2247
Super-2	0.0031	0.0009	1.65e$+$4	0.2163
Super-3	0.0032	0.0008	1.82e$+$4	0.2070
Super-4	0.0033	0.0007	2.04e$+$4	0.1963
Super-5	0.0034	0.0006	2.38e$+$4	0.1842
Super-6	0.0035	0.0005	2.74e$+$4	0.1702
Super-7	0.0036	0.0004	3.34e$+$4	0.1537
Super-8	0.0037	0.0003	4.35e$+$4	0.1336
Super-9	0.0038	0.0002	6.38e$+$4	0.1078
Super-10	0.0039	0.0001	1.30e$+$5	0.0696
Super-11	0.0040	0	1.63e$+$4	5.0e−4
Super-12	0.0041	-0.0001	5.99e$+$4	1.3e−4
Super-13	0.0042	-0.0002	3.86e$+$4	7.2e−5
Super-14	0.0043	-0.0003	2.90e$+$4	5.0e−5

Let us finally mention that the numerical scheme for the macroscopic model is amenable to parallelization since the numerical cost bottleneck is due to the convolution terms and this is not difficult to parallelize as it is scalable using load balancing between processors. The communication costs to compute convolutions are highly reduced due to the compact support of the potential. The other only communication costs will come at the time of evaluating the new values of the density that needs fluxes from neighboring cells. However, since we only use two numerical values of the flux, the communication costs will be rather small. These aspects from the parallel programming perspective have been treated in equations with similar issues such as kinetic equations, see, for instance, [28]. The strategy there can be applied directly to our situation leads to speedups for two-dimensional computations of the macroscopic model.

## Appendix 2: Tables

### Numerical Results for the One-Dimensional Case

It this section, we provide the data for the one-dimensional simulations of the supercritical bifurcation (Table 5) and of the subcritical bifurcation (Table 6) for the macroscopic model.

**Table 6:** Subcritical bifurcation in the one-dimensional case

$l_2=0.3$	$D_{2,\lambda }$	$\lambda$	$T_\mathrm{max}$	*Q*
Sub-1	0.0338	0.0010	0.54e$+$3	0.2926
Sub-2	0.0339	0.0009	0.57e$+$4	0.2921
Sub-3	0.0340	0.0008	0.61e$+$4	0.2915
Sub-4	0.0340	0.0007	0.61e$+$4	0.2915
Sub-5	0.0341	0.0006	0.67e$+$4	0.2909
Sub-6	0.0342	0.0005	0.74e$+$4	0.2903
Sub-7	0.0343	0.0004	0.83e$+$4	0.2897
Sub-8	0.0344	0.0003	0.98e$+$4	0.2891
Sub-9	0.0345	0.0002	1.26e$+$4	0.2884
Sub-10	0.0346	0.0001	2.03e$+$4	0.2878
Sub-11	0.0347	0	1.01e$+$5	2.2e−4
Sub-12	0.0348	-0.0001	4.98e$+$4	9.5e−5
Sub-13	0.0349	-0.0002	3.43e$+$4	6.1e−5
Sub-14	0.0350	-0.0003	2.66e$+$4	4.5e−5

### Numerical Results for the Two-Dimensional Case

It this section, we provide the data for the two-dimensional simulations of the supercritical bifurcation (Tables 7, 8) and of the subcritical bifurcation (Tables 9, 10, 11) for the macroscopic model.

**Table 7:** Supercritical bifurcation in the two-dimensional case: $\alpha =0.3$, $R/L=1$

Case 1	$D_{\lambda }$	$\lambda$	$T_\mathrm{max}$	*Q*
Super-1	0.7514	0.010	791.1	0.0111
Super-2	0.7523	0.009	871.5	0.0106
Super-3	0.7533	0.008	984.9	0.0099
Super-4	0.7542	0.007	1.1183e$+$3	0.0093
Super-5	0.7551	0.006	1.2971e$+$3	0.0086
Super-6	0.7560	0.005	1.5498e$+$3	0.0079
Super-7	0.7569	0.004	1.9324e$+$3	0.0070
Super-8	0.7578	0.003	2.5875e$+$3	0.0060
Super-9	0.7587	0.002	3.9821e$+$3	0.0048
Super-10	0.7596	0.001	9.2876e$+$3	0.0030
Super-11	0.7605	0.000	7.7450e$+$3	8.3456e-6
Super-12	0.7615	-0.001	2.6994e$+$3	2.0849e-6
Super-13	0.7624	-0.002	1.7778e$+$3	1.2446e-6
Super-14	0.7633	-0.003	1.3437e$+$3	8.8705e-7

**Table 8:** Supercritical bifurcation in the two-dimensional case: $\alpha =0.3$, $R/L=0.975$

Case 2	$D_{\lambda }$	$\lambda$	$T_\mathrm{max}$	*Q*
Super-1	0.7254	0.005	1.3932e$+$3	0.0102
Super-2	0.7264	0.004	1.8138e$+$3	0.0091
Super-3	0.7273	0.003	2.5098e$+$3	0.0078
Super-4	0.7282	0.002	4.1365e$+$3	0.0062
Super-5	0.7291	0.001	9.0104e$+$3	0.0042

**Table 9:** Subcritical bifurcation in the two-dimensional case: $\alpha =0.3$ $R/L=0.950$

Case 3	$D_{\lambda }$	$\lambda$	$T_\mathrm{max}$	*Q*
Sub-1	0.6923	0.005	1.1447e$+$3	0.0142
Sub-2	0.6932	0.004	1.4385+3	0.0133
Sub-3	0.6941	0.003	1.9591e$+$3	0.0123
Sub-4	0.6950	0.002	3.1522e$+$3	0.0109
Sub-5	0.6959	0.001	6.4408e$+$3	0.0093

**Table 10:** Subcritical bifurcation in the two-dimensional case: $\alpha =0.3$ $R/L=0.925$

Case 4	$D_{\lambda }$	$\lambda$	$T_\mathrm{max}$	*Q*
Sub-1	0.6570	0.005	858.4	0.0190
Sub-2	0.6579	0.004	1.0410e$+$3	0.0185
Sub-3	0.6588	0.003	1.3428e$+$3	0.0179
Sub-4	0.6597	0.002	1.9528e$+$3	0.0173
Sub-5	0.6607	0.001	4.6192e$+$3	0.0165

**Table 11:** Subcritical bifurcation in the two-dimensional case: $\alpha =0.3$ $R/L=0.900$

Case 5	$D_{\lambda }$	$\lambda$	$T_\mathrm{max}$	*Q*
Sub-1	0.6156	0.010	405.3	0.0244
Sub-2	0.6165	0.009	437.8	0.0241
Sub-3	0.6174	0.008	478.4	0.0239
Sub-4	0.6183	0.007	530.3	0.0236
Sub-5	0.6192	0.006	598.7	0.0233
Sub-6	0.6201	0.005	693.1	0.0230
Sub-7	0.6210	0.004	832.6	0.0226
Sub-8	0.6219	0.003	1.0628e$+$3	0.0223
Sub-9	0.6229	0.002	1.6089e$+$3	0.0219
Sub-10	0.6238	0.001	3.4882e$+$3	0.0215
Sub-11	0.6247	0.000	8.0028e$+$3	8.4646e−6
Sub-12	0.6256	-0.001	2.9256e$+$3	2.2626e−6
Sub-13	0.6265	-0.002	1.8733e$+$3	1.3058e−6
Sub-14	0.6274	-0.003	1.3983e$+$3	9.1773e−7

## Data Availability

No new data were collected in the course of this research.
